# Síntesis de evidencia y recomendaciones para el manejo de la suplementación con calcio antes y durante el embarazo para la prevención de la preeclampsia y sus complicaciones

**DOI:** 10.26633/RPSP.2021.134

**Published:** 2021-11-03

**Authors:** Organización Panamericana de la Salud

**Affiliations:** 1 Organización Panamericana de la Salud Washington D.C. Estados Unidos de América Organización Panamericana de la Salud, Washington D.C., Estados Unidos de América.

**Keywords:** Calcio, embarazo, preeclampsia, eclampsia, diagnóstico, tratamiento, Américas, Calcium, pregnancy, preclampsia, eclampsia, diagnosis, therapeutics, Americas, Cálcio, gravidez, preclampsia, eclampsia, diagnóstico, terapêutica, Américas

## Abstract

**Introducción.:**

La preeclampsia es una de las principales causas de morbimortalidad maternofetal en el mundo. La suplementación con calcio ha demostrado prevenir este trastorno y, por lo tanto, es importante contar con guías que emitan recomendaciones respecto de su uso.

**Objetivos.:**

Sintetizar las recomendaciones relacionadas con la preeclampsia desarrolladas por la Organización Mundial de la Salud (OMS) con el fin de mejorar la calidad del cuidado y desenlaces en salud de las mujeres en edad reproductiva y embarazadas, y abordar aspectos sobre su implementación.

**Métodos.:**

Las guías elaboradas por la OMS siguen los métodos de elaboración de las guías GRADE (*Grading of Recommendations Assessment Development and Evaluation*) del *Manual para la elaboración de directrices* de la OMS. Se llevó a cabo una síntesis de las recomendaciones de dos guías de la OMS. Además, se realizó una búsqueda sistemática en PubMed, Lilacs, Health Systems Evidence, Epistemonikos y literatura gris de estudios desarrollados en la Región de las Américas con el fin de identificar barreras, facilitadores y estrategias de implementación, así como determinar indicadores.

**Resultados.:**

Se formularon dos recomendaciones relacionadas con preeclampsia, eclampsia y sus complicaciones para aplicar antes y durante el embarazo. Se identificaron barreras, facilitadores para la implementación y se crearon indicadores de adherencia y resultado.

**Conclusiones.:**

Las recomendaciones formuladas buscan proveer orientación sobre cómo prevenir la preeclampsia a través del consumo de calcio con consideraciones para su implementación en América Latina y el Caribe.

## Abstract (en)

**Introduction.:**

Preeclampsia is one of the leading causes of maternal and fetal morbidity and mortality in the world. Calcium supplementation has proven to prevent this disorder. It is therefore important to issue guidelines and recommendations on its use.

**Objectives.:**

Synthesize the recommendations regarding preeclampsia developed by the World Health Organization (WHO) with a view to improving the quality of care and outcomes for the health of pregnant women and women of childbearing age in general, and address aspects of their implementation.

**Methods.:**

The guidelines prepared by WHO follow the GRADE method (Grading of Recommendations, Assessment, Development, and Evaluation) for the preparation of guidelines in the WHO Manual for the Preparation of Directives. Two of the WHO guidelines were synthesized and a systematic search was conducted in PubMed, Lilacs, Health Systems Evidence, Epistemonikos, and gray literature for studies carried out in the Region of the Americas in order to identify barriers, facilitators, and implementation strategies, as well as to determine indicators.

**Results.:**

Two recommendations related to preeclampsia and eclampsia and their complications were formulated for implementation before and during pregnancy. Implementation barriers and facilitators were identified and indicators were created for assessing adherence and outcomes.

**Conclusion.:**

The formulated recommendations aim to provide guidance on how to prevent preeclampsia through the consumption of calcium and include suggestions for implementation in Latin America and the Caribbean.

## RESUMO

**Introdução.:**

A pré-eclâmpsia é uma das principais causas de morbimortalidade materno-fetal no mundo. A suplementação com cálcio demonstrou prevenir esse transtorno e, portanto, é importante contar com guias que forneçam recomendações no que se refere ao seu uso.

**Objetivos.:**

Sintetizar as recomendações relacionadas à pré-eclâmpsia, desenvolvidas pela Organização Mundial da Saúde (OMS), a fim de melhorar a qualidade do cuidado e dos desfechos de saúde das mulheres em idade reprodutiva e grávidas, e abordar aspectos sobre sua implementação.

**Métodos.:**

Os guias elaborados pela OMS seguem os métodos de elaboração dos guias GRADE (do inglês, *Grading of Recommendations Assessment Development and Evaluation*) do *Manual para a elaboração de diretrizes* da OMS. Foi realizada uma síntese das recomendações de dois guias da OMS. Além disso, foi realizada uma busca sistemática nas bases PubMed, Lilacs, Health Systems Evidence e Epistemonikos e na literatura cinzenta de estudos conduzidos na Região das Américas para identificar barreiras, facilitadores e estratégias de implementação, bem como para definir indicadores.

**Resultados.:**

Foram formuladas duas recomendações relacionadas à pré-eclâmpsia, à eclâmpsia e a suas complicações para serem aplicadas antes e durante a gravidez. Foram identificadas barreiras e facilitadores para sua implementação e foram criados indicadores de adesão e resultado.

**Conclusões.:**

As recomendações formuladas buscam oferecer orientações sobre como prevenir a pré-eclâmpsia por meio do consumo de cálcio, com considerações para sua implementação na América Latina e no Caribe.

## INTRODUCCIÓN

La preeclampsia es una de las principales causas de morbilidad y mortalidad maternofetal en todo el mundo (1). Se considera hipertensión en el embarazo cuando la presión arterial sistólica es mayor o igual a 140 mmHg o la presión arterial diastólica es mayor o igual a 90 mmHg (2). Si bien los criterios clásicos para el diagnóstico de preeclampsia son la hipertensión y la proteinuria, existen otros criterios también importantes. Se considera que una mujer embarazada con hipertensión y sin proteinuria tiene preeclampsia cuando presenta cualquiera de las siguientes características: trombocitopenia (conteo de plaquetas menor de 100 000 × 10^9^/L), concentraciones elevadas de enzimas hepáticas, dolor epigástrico grave y persistente que no obedece a otros diagnósticos, insuficiencia renal, edema pulmonar o cefalea que no responde a analgésicos y no se debe a otros diagnósticos (3). Entre los factores de riesgo más frecuentes se asocian la nuliparidad, el embarazo múltiple, antecedentes de hipertensión crónica, la diabetes mellitus y la edad materna de 35 años o más. El riesgo de preeclampsia aumenta según el índice de masa corporal (IMC) previo al embarazo (3–5). La preeclampsia se puede clasificar como no grave o grave (6). La preeclamspia grave se define por la presencia de daño en uno o más sistemas: cuando las cifras de tensión arterial son mayores de 160/110 mmHg o existe edema pulmonar o deterioro de la función renal o hemólisis, enzimas hepáticas elevadas y bajo recuento de plaquetas. Esta constelación de signos y síntomas se conoce como síndrome HELLP (por su sigla en inglés de hemólisis, elevación de las enzimas hepáticas y plaquetopenia), presente en el 10-20% de las mujeres con preeclampsia grave (7).

La mayoría de las muertes relacionadas con trastornos hipertensivos pueden evitarse si se proporciona educación y atención oportuna a las mujeres que presentan tales complicaciones. La Organización Mundial de la Salud (OMS) ha recomendado indicar un complemento con calcio a las mujeres embarazadas, especialmente para la población de alto riesgo, combinado con una dieta con bajo contenido de calcio (8).

Una revisión sistemática identificó los efectos de la suplementación con calcio y vitamina D sobre la preeclampsia y el riesgo de presentar hipertensión gestacional o hipertensión inducida por el embarazo, y se sugiere que la suplementación con calcio podría causar una reducción significativa del riesgo de preeclampsia, hipertensión gestacional o hipertensión inducida por el embarazo en aproximadamente 50% y 25% de los casos, respectivamente, en comparación con la administración de placebo (9). Otra revisión sistemática identificó que la suplementación con bajas dosis de calcio causó una reducción en la incidencia de preeclampsia, con un riesgo relativo (RR) de 0,36 (intervalo de confianza del 95% [IC95%]:0,23-0,57); en otro grupo, la suplementación con calcio con o sin suplementos adicionales informó un RR de 0,38 (IC95%: 0,28-0,52); la suplementación de calcio en dosis bajas más el ácido linoleico un RR de 0,23 (IC95%: 0,09-0,60), y la suplementación de calcio en dosis bajas más vitamina D un RR de 0,49 (IC95%: 0,31-0,78) (10).

Con base en estas consideraciones, se vuelve necesario establecer los lineamientos para indicar la suplementación con calcio en mujeres, en particular: 1) aquellas que pretenden quedar embarazadas, 2) aquellas con mayor riesgo de trastornos hipertensivos asociados al embarazo, y 3) embarazadas, para la prevención de preeclampsia y sus complicaciones.

### Objetivos y población diana

Las guías para la suplementación con calcio antes y durante el embarazo para la prevención de la preeclampsia y sus complicaciones tienen como objetivo mejorar la calidad del cuidado y los desenlaces en salud de las mujeres embarazadas, en especial aquellos relacionados con preeclampsia, eclampsia y sus complicaciones (11,12).

### Alcance y usuarios

Las guías de práctica clínica proveen recomendaciones informadas en la evidencia para las mujeres, en particular para: 1) aquellas que pretenden quedar embarazadas, 2) aquellas con mayor riesgo de trastornos hipertensivos asociados al embarazo, y 3) embarazadas en países de bajos, medianos y altos ingresos y quienes viven en zonas donde la ingesta de calcio es baja. Las recomendaciones están dirigidas a profesionales de la salud (ginecólogos obstetras, enfermeras, nutricionistas y médicos generales) (11,12).

Se pretende que las recomendaciones sean usadas por los responsables de tomar decisiones a nivel individual, institucional u organizacional y los miembros de entidades gubernamentales, con el fin de facilitar el proceso de implementación.

## MÉTODOS

### Desarrollo de las recomendaciones

Para elaborar las recomendaciones se siguieron los métodos del manual para la elaboración de directrices de la OMS (13). Los pasos desarrollados fueron: 1) identificación de preguntas clínicas y desenlaces, 2) búsqueda de la evidencia, 3) evaluación y síntesis de la evidencia, 4) formulación de recomendaciones incluidas las prioridades de investigación, 5) diseminación, y 6) consideraciones éticas, de equidad y de implementación. Las recomendaciones fueron elaboradas siguiendo los lineamientos de GRADE (14) y se prepararon los perfiles de evidencia con base en revisiones sistemáticas actualizadas (7). Se utilizaron el enfoque DECIDE (15) (por su sigla en inglés de *Decisions and Practice based on Evidence*) y los marcos de evidencia para la decisión (ETD, por su sigla en inglés) para orientar las recomendaciones con base en la calidad de la evidencia, el efecto de las intervenciones, los recursos, la equidad, la aceptabilidad y la factibilidad (11,12).

La información sobre el detalle metodológico y la evidencia que apoya las recomendaciones se puede encontrar en la versión larga de las guías (11,12).

### Cómo usar las recomendaciones

Cada recomendación presenta la calidad de la evidencia siguiendo el sistema GRADE (14):

**Table:** 

**Criterio clínico**	**Características**
**Alta ⊕⊕⊕⊕**	Es muy poco probable que nuevos estudios cambien la confianza que se tiene en el resultado estimado.
**Moderada ⊕⊕⊕○**	Es probable que nuevos estudios tengan un impacto importante en la confianza que se tiene en el resultado estimado y que estos puedan modificar el resultado.
**Baja ⊕⊕○○**	Es muy probable que nuevos estudios tengan un impacto importante en la confianza que se tiene en el resultado estimado y que estos puedan modificar el resultado.
**Muy baja ⊕○○○**	Cualquier resultado estimado es muy incierto.

Las recomendaciones incluyen las siguientes categorías:

**Table:** 

**Fuerza de la recomendación**	**Significado**
Fuerte	Las consecuencias deseables claramente sobrepasan las consecuencias indeseables. **SE RECOMIENDA HACERLO**
Condicional	Las consecuencias deseables probablemente sobrepasan las consecuencias indeseables. **SE SUGIERE HACERLO**
Recomendado solo en el contexto de investigación rigurosa	La implementación aún puede llevarse a cabo a gran escala, siempre que la misma adopte la forma de una capaz de abordar las incertidumbres relacionadas tanto con la eficacia de la intervención u opción, y su aceptabilidad y viabilidad.

### Desarrollo de la síntesis de evidencia

Se sintetizó la información de las guías para el manejo de la suplementación con calcio antes y durante el embarazo para la prevención de la preeclampsia y sus complicaciones relacionada con la metodología, el alcance, los objetivos, el resumen de las recomendaciones y la calidad de la evidencia mediante el uso de un formato predeterminado. Se realizaron búsquedas con el objetivo de identificar estudios que abordaran aspectos de implementación (barreras, facilitadores, estrategias de implementación e indicadores) usando la estrategia de búsqueda de la guía y filtros para identificar estudios sobre consideraciones para la implementación (16). La estrategia de búsqueda incluyó los términos “adoption, uptake, utilization; taken implementation, dissemination, evidence-based treatment, barriers”. La búsqueda se realizó en Pubmed, LILACS, Health Systems Evidence y Epistemonikos hasta marzo de 2021. No se realizó evaluación de la calidad de la evidencia incluida. Se seleccionaron revisiones sistemáticas y estudios primarios con el objetivo de identificar las consideraciones para la implementación de las recomendaciones de la guía. Estas se organizaron de acuerdo con el tipo de barrera (vinculadas a factores del sistema de salud, organizacionales o estructurales; barreras relacionadas con las mujeres embarazadas; conocimiento de la guía; recursos financieros, materiales y tecnológicos; y acceso). Para las barreras identificadas, se seleccionaron los facilitadores y las estrategias de implementación más efectivas considerando el contexto de la Región. A partir de la literatura seleccionada, se identificaron y construyeron indicadores de proceso y de resultado de implementación de la guía. Por último, un grupo interdisciplinario de metodólogos y expertos temáticos de la Organización Panamericana de la Salud (OPS) revisó los aspectos relacionados con la implementación.

## RECOMENDACIONES

En el cuadro 1 se presentan las recomendaciones y las consideraciones para la suplementación con calcio antes y durante el embarazo para la prevención de la preeclampsia y sus complicaciones (11,12).

### Implementación

Se recomienda que los siguientes actores apoyen la implementación de las recomendaciones (11,12): ginecólogos obstetras, enfermeras, nutricionistas y médicos generales; direcciones del sistema nacional de salud (SNS) de cada país; entidades gubernamentales; instituciones universitarias; administrativos de instituciones que brindan atención primaria; y actores clave de los sistemas de salud de cada país.

**CUADRO 1.** Recomendaciones para la suplementación con calcio antes y durante el embarazo para la prevención de la preeclampsia y sus complicaciones

**Categoría**	**Recomendación**
Suplementación con calcio durante el embarazo para la prevención de la preeclampsia y sus complicaciones	
Fuerte	En poblaciones con bajo consumo dietario, se recomienda la suplementación con calcio (1,5-2 g de calcio elemental oral) para mujeres embarazadas para reducir el riesgo de preeclampsia. **Calidad de la evidencia: moderada**
Consideraciones Esta recomendación es congruente con las recomendaciones del 2016 de la OMS sobre el cuidado prenatal para experimentar un embarazo positivo. En la consulta dietaria de mujeres embarazadas, se debe promover la ingesta adecuada de calcio a través de comidas con alto contenido de calcio disponibles localmente. Se deben evaluar las barreras, los facilitadores y los algoritmos para integrar la recomendación dentro del paquete de cuidado prenatal. Dividir la dosis de calcio puede mejorar la aceptabilidad. La dosis sugerida para la suplementación con calcio es 1,5-2 g/día, que se puede dividir en tres dosis, preferiblemente con las comidas. Pueden presentarse interacciones negativas entre el hierro y el calcio. Por lo tanto, los suplementos deben administrarse a diferentes horas y no de manera simultánea. No existe evidencia acerca del tiempo de inicio de la suplementación con calcio, los profesionales de la salud pueden iniciar la suplementación en la primera visita prenatal con el fin de mejorar la adherencia a la recomendación. Con el fin de llegar a las poblaciones vulnerables o asegurar una administración continua y oportuna de los suplementos, los grupos de interés pueden considerar una reasignación de la provisión de la suplementación con calcio en las comunidades con poco acceso a profesionales de la salud. Se deben monitorear la implementación y el impacto de la recomendación en los servicios de salud a nivel nacional y regional, según los criterios definidos y los indicadores asociados con una población diana acordada a nivel local. La evaluación local de las barreras y los facilitadores existentes aumentará las posibilidades de integrar la recomendación dentro del paquete de cuidado prenatal.	
Suplementación con calcio antes del embarazo para la prevención de la preeclampsia y sus complicaciones	
Solo en el contexto de una investigación rigurosa	La administración de suplementos con calcio antes del embarazo para la prevención de la preeclampsia y sus complicaciones se recomienda solo en el contexto de una investigación rigurosa. **Calidad de la evidencia: baja**
Consideraciones Se desconoce aún si el inicio de la administración de suplementos con calcio antes del embarazo y su continuación durante este conferirá beneficios adicionales para la salud, por lo que se requieren nuevas investigaciones. La fortificación de alimentos con calcio puede ser una intervención importante de salud pública en entornos donde la ingestión de calcio con la dieta es baja. El consejo dietético a todas las mujeres que estén considerando el embarazo debe promover una ingestión adecuada a través de alimentos con alto contenido de calcio localmente disponibles. Se podría lograr con facilidad una ingestión adecuada de calcio mediante el consumo diario de productos lácteos. Sin embargo, los productos lácteos no forman parte de todas las dietas, o no están disponibles en ciertas poblaciones. Asimismo, una dieta con alto contenido de sal reduce la retención de calcio corporal, en comparación con una dieta con bajo contenido de sal. La cafeína y las proteínas también pueden inducir hipercalciuria, pero en mucha menor medida. Esto se ha vuelto más importante en los últimos años debido al consumo de bebidas que contienen cafeína, como los refrescos y las bebidas energéticas.	

Dentro del proceso de implementación, es determinante identificar las posibles barreras, los facilitadore y las estrategias para mejorar el cumplimiento de las recomendaciones. En el cuadro 2 se presentan algunas barreras, facilitadores y estrategias de implementación que pueden ser consideradas por los países (11,12,16–23).

En el recuadro 1 se sugieren los indicadores de proceso y resultado de la implementación de la guía, en base a la revisión de la literatura de esta síntesis y las consideraciones del panel de expertos de la Región.

**CUADRO 2.** Barreras, facilitadores y estrategias de implementación de la suplementación con calcio antes y después del embarazo

**Aspectos**	**Barreras**	**Facilitadores**	**Estrategias de implementación**
Vinculados a factores del sistema de salud, organizacionales o estructurales	Desconocimiento de las zonas donde la ingestión de calcio es baja Desconocimiento de los métodos para medir la ingestión de calcio	Proveedores de servicios de salud, sociedades científicas y universidades	Realizar capacitaciones a los profesionales de la salud de los proveedores de salud sobre las recomendaciones de las guías con el fin de proveer la suplementación con calcio a las madres embarazadas Capacitar a los profesionales designados para medir la ingestión de calcio en la población (encuestas dietéticas utilizando diarios de 24 horas, cuestionarios de frecuencia alimentaria o pesaje de alimentos) Promover el uso de datos secundarios derivados de hojas de balance de alimentos o encuestas de consumo a hogares de la Organización de las Naciones Unidas para la Agricultura y la Alimentación
Barreras relacionadas con las mujeres embarazadas	Las mujeres informan que no conocen cuáles son los alimentos con alto contenido de calcio La adherencia a la recomendación del consumo de calcio o los suplementos puede ser baja (ej. Eventos adversos) Las mujeres pueden decidir usar calcio previo al embarazo sin orientación de un profesional de la salud	Entidades gubernamentales Proveedores de servicios de salud Medios públicos de comunicación	Se debe brindar información a las mujeres sobre qué alimentos con alto contenido de calcio deben consumir y en qué momento del embarazo deben hacerlo Desarrollo de recordatorios telefónicos para mantener la ingestión de alimentos necesaria para cumplir con los requerimientos de calcio Dividir la dosis de calcio en tres dosis, preferiblemente con las comidas, para mejorar la aceptabilidad Iniciar la suplementación en la primera visita prenatal, con el fin de mejorar la adherencia a la recomendación Las mujeres embarazadas y su entorno deben recibir información transparente de los beneficios y los perjuicios esperados de la ingestión de calcio o suplementos y del impacto de la preeclampsia y la eclampsia sobre la salud maternofetal Las mujeres deben recibir asesoramiento adecuado sobre los beneficios del uso del calcio antes del embarazo y sus posibles efectos secundarios
Conocimiento de la guía de práctica clínica	Los profesionales de la salud no conocen las recomendaciones sobre la suplementación con calcio en mujeres embarazadas para la prevención de la preeclampsia Los profesionales no conocen la recomendación para el uso en el contexto de la determinación de los niveles de calcio antes del embarazo	Proveedores de servicios de salud Entidades gubernamentales Sociedades científicas Profesionales de salud dispuestos a analizar de manera temprana la evidencia, monitorizar la práctica y los procesos	Socializar ambas recomendaciones a los profesionales de salud sobre dónde encontrar las guías en los proveedores de salud Alojar las guías en las páginas web de los repositorios nacionales de guías: páginas web de entidades gubernamentales, sociedades científicas y hospitales Realizar recordatorios en las historias clínicas sistematizadas Difusión en revistas, boletines, aplicaciones móviles y códigos QR
Recursos financieros, materiales y tecnológicos	Dificultades en la provisión de calcio por problemas en la cadena de abastecimiento Dificultad para diseñar y realizar estudios aleatorizados en mujeres antes del embarazo para reducir la incertidumbre acerca del impacto de los suplementos en esta población en relación con el riesgo de preeclampsia y eclampsia Recursos financieros limitados destinados a los procesos de capacitación (teórica y práctica) en el uso de la guía de práctica clínica Costos “de bolsillo” elevados de los alimentos con alto contenido de calcio y los suplementos	Entidades gubernamentales Proveedores de servicios de salud Administradores de establecimientos de salud que tienen farmacias internas	Disponibilidad de suplementos con calcio en las instituciones que proveen cuidado prenatal Considerar una reasignación de la provisión de la suplementación con calcio en comunidades con poco acceso a profesionales de la salud Favorecer el desarrollo de políticas que optimicen la fortificación con calcio de los alimentos Cada país revisará la inclusión de calcio en su plan de beneficios Desarrollar estudios no aleatorizados controlados (p. ej. series de tiempo interrumpido o estudios de antes y después) con la mayor similitud posible entre aquellos expuestos y no expuestos a los suplementos con calcio para conocer el impacto del suplemento antes del embarazo Utilizar reuniones científicas y espacios en cursos de capacitación en la Región para capacitar en forma teórica y práctica sobre el uso correcto de la guía Fortalecer las políticas que permitan el financiamiento de los procesos de capacitación (teórica y práctica) en el uso de la guía de práctica clínica
Acceso	Dificultad para asistir a los controles prenatales Factores relacionados con el sistema de salud, organizacionales o estructurales	Prestadores de atención de salud y sus instituciones Personal y técnicos que trabajan en los servicios de salud	Talleres de capacitación continua para el personal de salud de los ámbitos público y privado sobre la organización de los servicios de salud, con especial foco en el control prenatal Establecer la infraestructura para unas líneas de acceso directo para el control prenatal (p. ej., implementación de servicios de telemedicina)

RECUADRO 1. **Indicadores de impacto y proceso en la implementación de la guía para el manejo de la suplementación con calcio antes y durante el embarazo para la prevención de la preeclampsia y sus complicaciones**

**Indicador de impacto**

- Proporción de mujeres embarazadas que desarrollan preeclampsia y eclampsia.

**Indicadores de proceso**

- Proporción de mujeres embarazadas que reciben suplementación con calcio dentro del paquete de servicios prenatal.
- Proporción de mujeres que consumen entre 1,5 y 2 g de calcio por día.

### Conclusiones

La OPS pone a disposición de los gerentes de servicios de salud y del personal de la salud una síntesis sobre las recomendaciones informadas en la evidencia y buenas prácticas para la suplementación con calcio para las mujeres antes y durante el embarazo, con el fin de orientar su uso de forma eficaz y segura y como una herramienta de implementación. Asimismo, se presentan algunas barreras para la implementación de las recomendaciones (p. ej., falta de conocimiento de la guía, desconocimiento de dónde encontrar alimentos con alto contenido de calcio, dificultades en la provisión de calcio por problemas en la cadena de abastecimiento y costos de los suplementos, entre otras) y estrategias para abordarlas (p. ej., fortalecimiento de políticas nacionales y capacitación para las madres y el personal de salud) , así como indicadores de proceso y resultado. Esta síntesis de evidencia busca favorecer la diseminación y el uso de las guías que elabora la OPS y contribuir a mejorar la calidad de la atención y la salud de la población de las embarazadas en la Región de las Américas.
